# Impact of inclusion of post-spermatic ejaculate fraction in boar seminal doses on sperm metabolism, quality, and interaction with uterine fluid

**DOI:** 10.1038/s41598-023-42254-3

**Published:** 2023-09-14

**Authors:** Chiara Luongo, Pedro José Llamas-López, Gabriela Garrappa, Ernesto Rodríguez-Tobón, Paulina Grudzinska, Francisco Alberto García-Vázquez

**Affiliations:** 1 Departamento de Fisiología, Facultad de Veterinaria, Campus de Excelencia Mare Nostrum, Universidad de Murcia, Murcia, Spain; 2 Departamento de Tecnología Agroalimentaria, Universidad Miguel Hernández, Elche, Spain; 3 Instituto de Investigación Animal del Chaco Semi-Arido (IIACS), Centro de Investigación Agropecuaria (CIAP), Instituto Nacional de Tecnología Agropecuaria (INTA), Tucuman, Argentina; 4 Departamento de Biología de La Reproducción, Universidad Autónoma Metropolitana, Unidad Iztapalapa, Ciudad de México, México; 5 Instituto Murciano de Investigación Biosanitaria (IMIB-Arrixaca), Murcia, Spain

**Keywords:** Physiology, Reproductive biology

## Abstract

Boar ejaculate is composed of sperm cells and seminal plasma (SP) and is emitted in different fractions (pre-sperm fraction; spermatic-rich fraction; intermediate fraction; post-spermatic fraction), with different composition of SP and volume, which could influence the sperm quality during seminal doses preparation, conservation, and interaction with the female reproductive tract. In artificial insemination (AI) centers, seminal doses are usually prepared with the spermatic-rich and intermediate fractions, but the inclusion of other ejaculate fractions, although controversial, is beginning to be applied. The objective was to evaluate the synergic effect of accumulative ejaculated fractions on sperm functionality during seminal doses preparation, throughout storage and after incubation with uterine fluid (UF). For this purpose, a total of 57 ejaculates were collected, and the following experimental groups were prepared (n = 19 per group): (F1) spermatic-rich fraction; (F2) F1 plus intermediate fraction; (F3) F2 plus post-spermatic fraction. Each group was stored for 5 days at ∼16 °C, and the following parameters were evaluated: sperm metabolism of pure and diluted semen (day 1), sperm quality parameters (days 1, 3, 5), thermal-resistance test (TRT) and incubation with uterine fluid (UF) (day 5). Sperm metabolic rates between accumulative ejaculate fractions from pure and diluted semen did not show differences. Also, sperm quality parameters were not affected by the ejaculate fraction during storage. However, sperm subjected to TRT showed similar results except for progressive motility, which was better in F2 and F3 than F1. When sperm were incubated with UF, the quality decreased in each group, but sperm from F2 and F3 were less affected than those from F1. In conclusion, the post-spermatic fraction can be included in seminal doses for their use in AI-centers, with functionality of sperm of different SP origins not being impaired throughout the storage, and responding better to thermal and UF stress. However, further research in AI-centers is necessary to test the sperm behaviour under presented conditions.

## Introduction

Over the years, the swine industry has been one of the most important agriculture-related businesses around the globe^1^. Most pigs are produced by artificial insemination (AI), a widespread reproductive technique, currently used in 90% of farms^2^. In fact, the boar ejaculate has a high volume (200–300 ml) with a concentration of ~ 30 × 10^9^ sperm/ml^3^, allowing to prepare numerous doses and bringing an economic advantage to AI-centers and farms. The ejaculate, composed of sperm cells and seminal plasma (SP), is emitted in different fractions: the first one, the pre-spermatic fraction, is composed of urethral content and secretions from bulbourethral glands and prostate, but usually without sperm cells; the second one, the spermatic-rich fraction, contains most of the sperm cells and SP (derived from testes, epididymis, seminal vesicles and prostate); the third one, the intermediate spermatic fraction, is considered a transition phase, containing lower sperm cell number and SP than spermatic-rich fraction; and finally, the fourth and last one, the post-spermatic fraction, contains few sperm cells and a high volume of SP (derived from seminal vesicles, prostate and bulbourethral glands)^4,5^. The common semen collection method has been the manual or gloved-hand method, with which only the rich fraction was collected for seminal doses preparation, avoiding mixing the SP from the subsequent fractions of the ejaculate^6^. In fact, by collecting the bulk ejaculate, sperm are mixed with the SP, proceeding from all of the ejaculated fractions at the same time, not respecting the physiological conditions, which explains the controversial effect of SP on sperm functionality^7^. Nevertheless, automatic methods are being used more frequently because of their efficiency and reduced time-consuming^8^. These methods do not allow to discern between fractions, so the bulk ejaculate is collected. This fact became the subject of discussion since the role of the SP and its components (which depend on the ejaculate fractions included)^9^ during storage is not fully elucidated. In this respect, a previous study^4^ showed similar reproductive performance (piglet health and development between sows inseminated with seminal doses prepared by using only the spermatic-rich fraction or the whole ejaculate and stored for 3 days). Sperm functionality during storage is gradually diminishing as the extended storage period increase (reviewed by^10^), although this fact has not yet been evaluated using the synergic effect of SP from accumulative ejaculate fractions during a period of storage beyond 3 days^4^, which is the time farms sometimes are required to store seminal doses for.

From ejaculation until seminal doses preparation and conservation, sperm are exposed to different environments. During this process, sperm functionality is influenced by several factors such as substrates (coming from SP and commercial extender), which can cause changes in sperm metabolism^11^. Energetic metabolism consists of the production of adenosine triphosphate (ATP) from the oxidation of biological molecules to simpler ones^11^. Specifically, sperm can obtain energy by two pathways: glycolysis and/or oxidative phosphorylation (OXPHOS), processes that take place in the principal piece and the midpiece (mitochondria electron transport chain) respectively (reviewed by^12^). In the case of porcine species, sperm were thought to use more glycolysis than OXPHOS (reviewed by^12^. However, research done by Nesci et al., 2020 demonstrated a correlation between sperm motility and mitochondrial activity, two functions dependent on OXPHOS, highlighting that boar sperm need ATP produced by this pathway to support their functionality. Although sperm mitochondria produce maximum energy in physiological conditions, this production of ATP by sperm cells may be affected by the environment, being able to switch the metabolic pathway depending on the substrates available^14^. In fact, SP composition differs between ejaculate fractions for proteins (mainly present in SP from the rich fraction), lipids, and metabolites that adhere to sperm surface and influence sperm functionality during storage and within the female genital tract^15–17^.

When seminal doses are deposited within the female reproductive tract and reach the uterus, SP plays a two-fold role. On one side SP protects spermatozoa from the inflamed uterine environment, helping with the maintenance of sperm motility and acrosome integrity, as shown in mouse and pig^18,19^. On the other side, SP regulates the immune response by SP immunoregulatory components interacting with the uterine environment^20^. Moreover, within the uterus, sperm encounter a complex female reproductive fluid-uterine fluid (UF), where an interaction between sperm and proteins proceeded from SP and UF is established^16^. Nevertheless, the effect of UF on boar sperm subjected to different SP fractions still needs to be elucidated. Based on the current knowledge, this effect could depend on the protein composition which varies between the fractions^9^. For instance, the spermatic-rich fraction is abundant in AWN spermadhesins involved in preventing early capacitation, allowing sperm to reach the site of fertilization^21^. Besides, the post-spermatic fraction is rich in PSP-I/PSP-II heterodimer^22^, a spermadhesin also involved in the prevention of sperm capacitation and immune response inducing the reduction of polymorphonuclear granulocytes within the female tract^16,23^.

Since sperm are subjected to a milieu conditioned by differential composition of ejaculate fractions, their behavior may be modified during seminal doses production and conservation, before being used in AI programs, and during sperm interaction with the female environment. Therefore, considering the important role of SP from each ejaculate fraction, the aim of the study was to evaluate the behavior of sperm from seminal doses prepared with different accumulative ejaculate fractions on metabolism, storage for 5 days, and incubation with UF.

## Results

**Figure 1:**
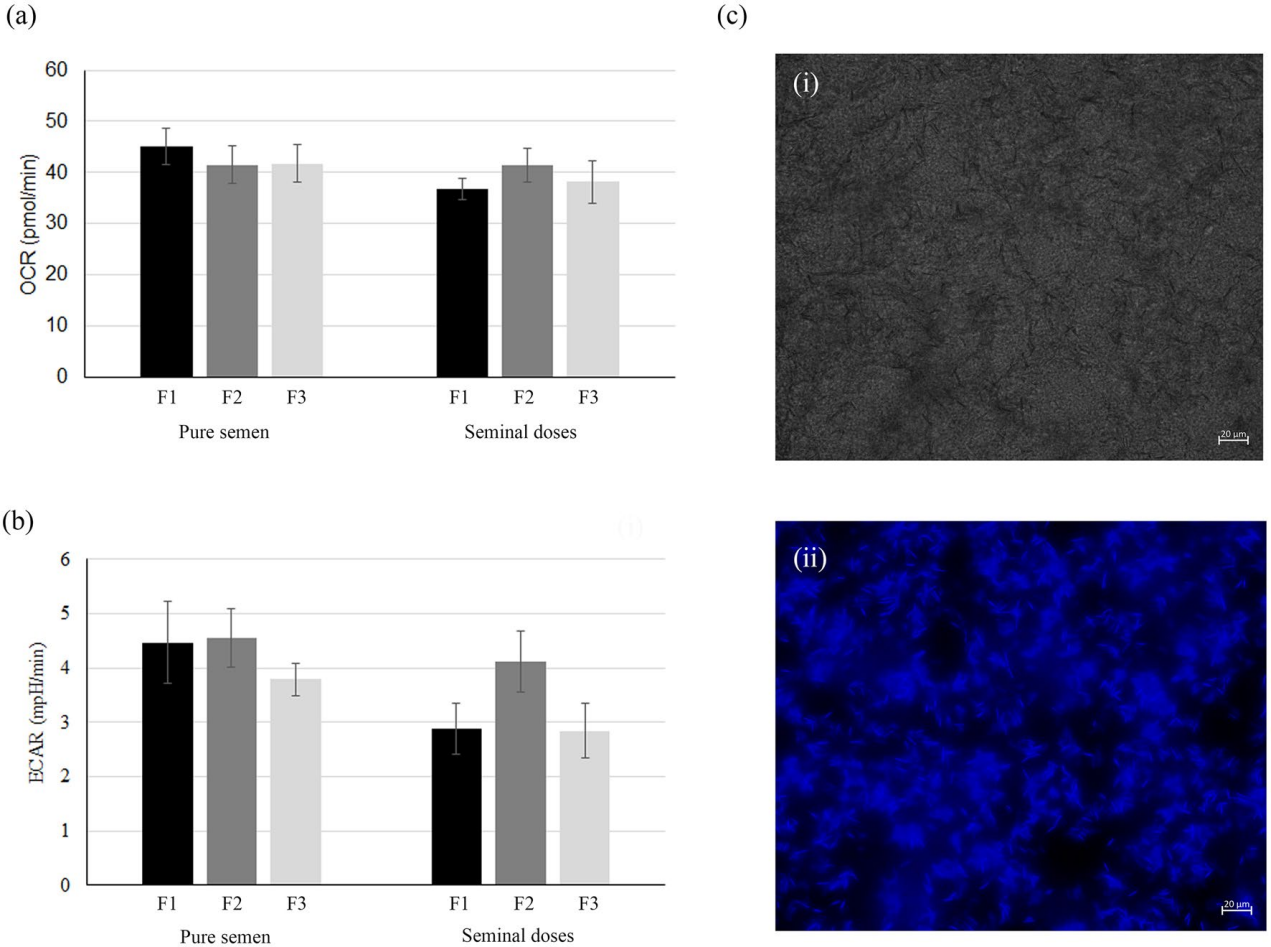
Analysis of sperm metabolism. (**a**) Oxygen Consumption Rate (OCR, pmol/min), and (**b**) Extracellular Acidification Rate (ECAR, mpH/min) (mean ± SEM) of spermatozoa from different accumulative ejaculated fractions (pure semen and seminal doses) at day 1: F1 (spermatic-rich fraction); F2 (F1 plus intermediate fraction); F3 (F2 plus post-spermatic fraction). (**c**) Images depicting spermatozoa attachment to the bottom of a well of the 96-wells plate, before metabolic measurements (i) and spermatozoa stained with Hoechst after metabolic measurements (ii). No statistical differences were observed between the groups (*P* > 0.05).

**Figure 2:**
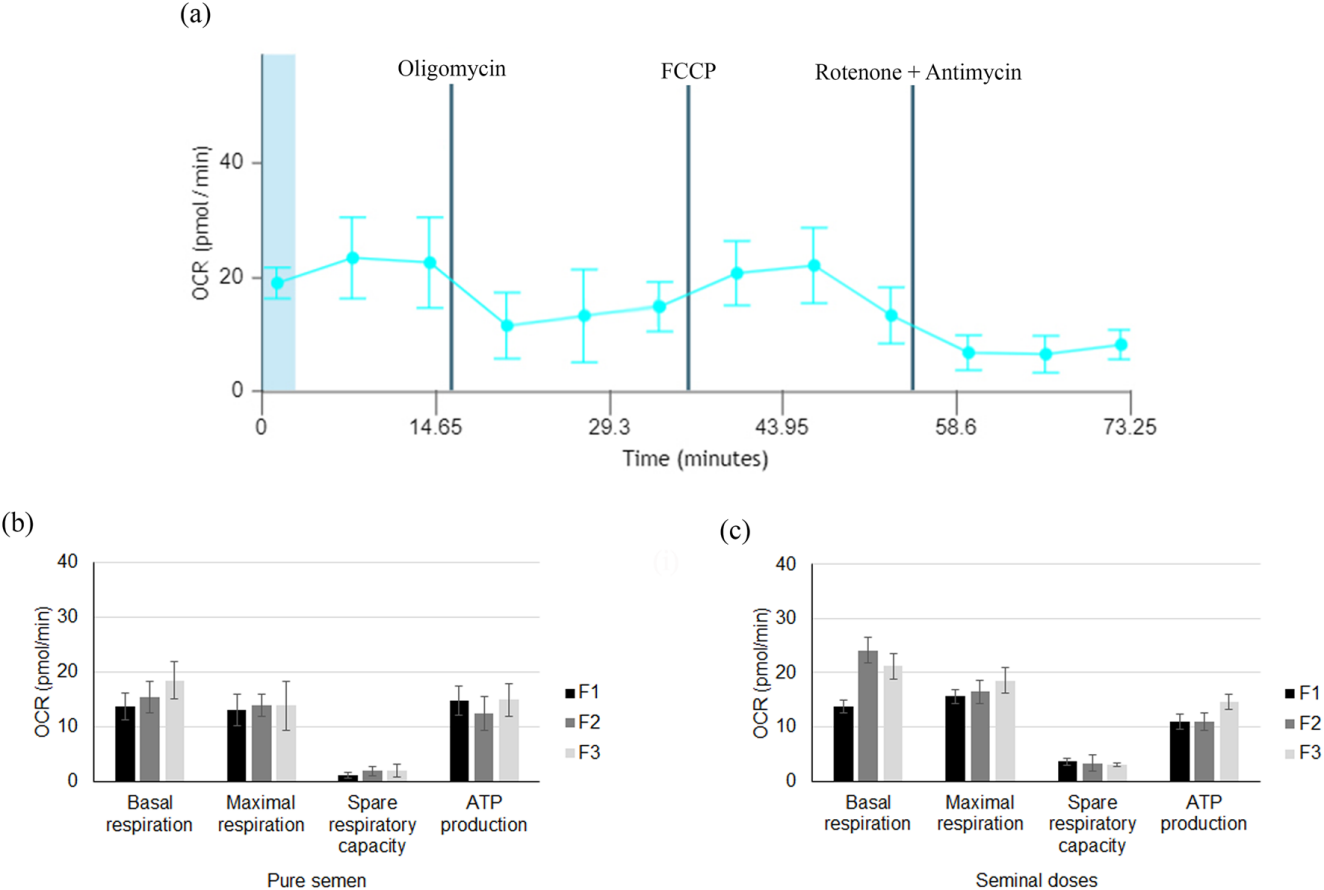
Analysis of sperm respiration. (**a**) An example of Oxygen Consumption Rate (OCR, pmol/min) analysis indicating basal respiration, ATP-linked respiration, and maximal respiration. (**b**) and (**c**) represent basal respiration, maximal respiration, spare respiratory capacity, and ATP production of spermatozoa from different accumulative ejaculated fractions (pure semen and seminal doses) at day 1: F1 (spermatic-rich fraction); F2 (F1 plus intermediate spermatic fraction); F3 (F2 plus post-spermatic fraction). No statistical differences were observed between groups (*P* > 0.05).

After semen collection and seminal doses preparation, sperm metabolism (Oxygen Consumption Rate-OCR and Extracellular Acidification Rate-ECAR) from pure and diluted semen of the 3 ejaculate fractions was analyzed. The OCR of sperm was not influenced by the fraction of the ejaculate neither in pure semen (F1 = 45.15 ± 3.57 pmol/min; F2 = 41.53 ± 3.75 pmol/min; F3 = 41.80 ± 3.70 pmol/min) (*P* = 0.720) nor in seminal doses (F1 = 36.80 ± 2.15 pmol/min; F2 = 41.35 ± 3.28 pmol/min; F3 = 38.15 ± 4.11 pmol/min) (*P* = 0.791) (Fig. 1a). Also, ECAR did not show significant difference neither for pure semen (F1 = 4.38 ± 0.78 mpH/min; F2 = 4.55 ± 0.54 mph/min; F3 = 3.79 ± 0.30 mpH/min) (*P* = 0.673) nor for seminal doses (F1 = 2.89 ± 0.47 mpH/min; F2 = 4.12 ± 0.56 mpH/min; F3 = 2.84 ± 0.50 mpH/min) (*P* = 0.378) (Fig. 1b). Moreover, when each corresponding group was compared with its counterpart (pure semen vs. seminal doses), no significant differences were observed neither for OCR (*P* > 0.05) nor ECAR (*P* > 0.05). The Seahorse analyzer also allowed to calculate basal and maximum respiration, spare respiratory capacity, and ATP production. No significant differences were shown for these parameters between the groups, nor when each pure sample was compared with its corresponding seminal doses (*P* > 0.05) (Fig. 2).

**Figure 3:**
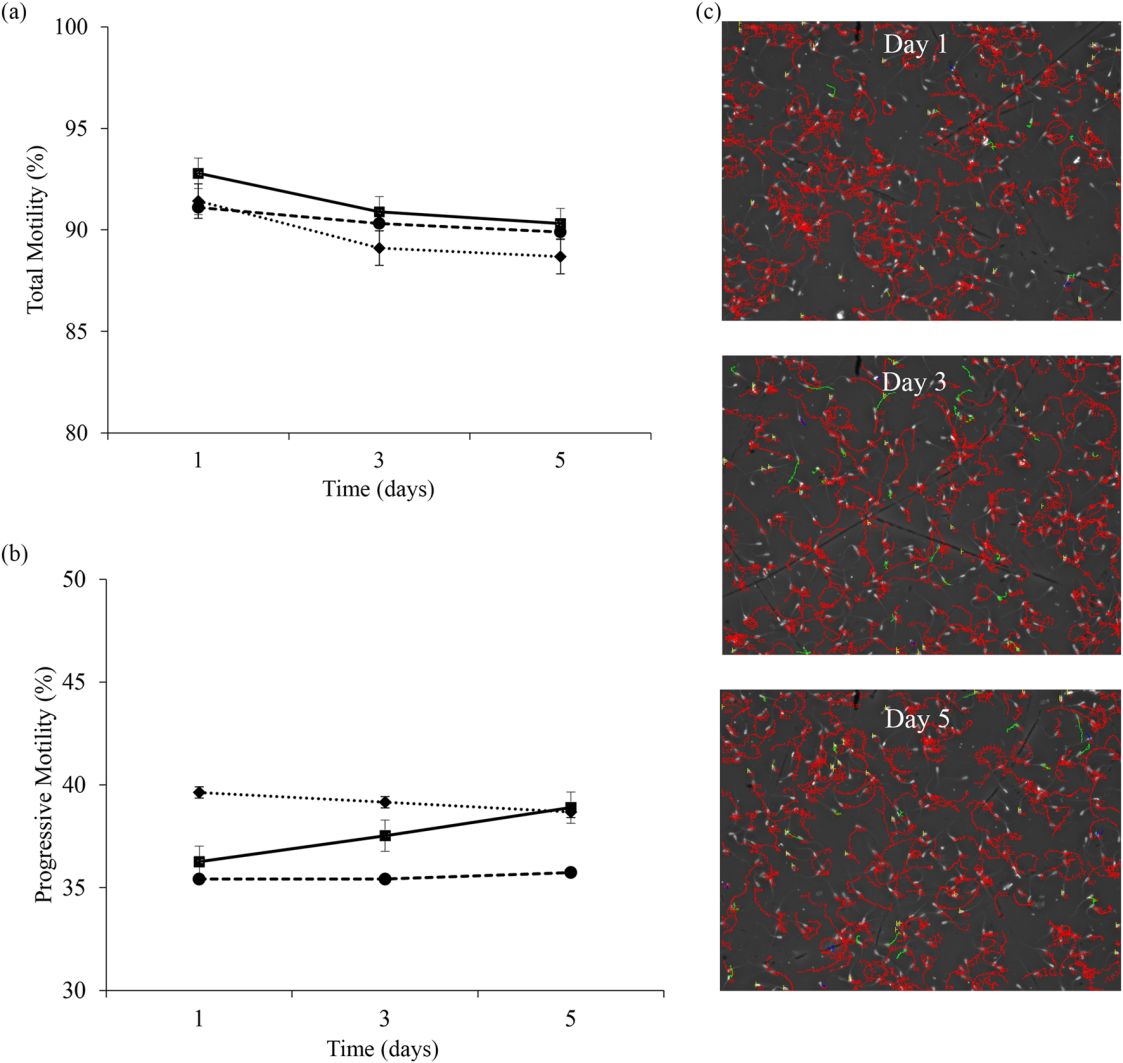
Analysis of sperm motility during storage. (**a**) Total motility (%) and (**b**) progressive motility (%) (mean ± SEM) of spermatozoa from different accumulative ejaculate fractions stored for 5 days (analyzed at days 1, 3, and 5) at a refrigeration temperature of ∼16 °C: F1 (spermatic-rich fraction) (filled square); F2 (F1 plus intermediate spermatic fraction) (filled circle); F3 (F2 plus post-spermatic fraction) (filled daimond). (**c**) Spermatozoa trajectory sequences obtained by CASA system on days 1 (upper image), 3 (middle image) and 5 (down image). The colors of sperm trajectories indicate fast velocity (red; > 45 µm/s), medium velocity (green; > 25–45 µm/s), low velocity (blue; 10–25 µm/s) or static sperm (yellow; < 10 µm/s).

Semen characteristics during preservation (evaluated on days 1, 3 and 5, and analyzed by repeated measures) are reported in Fig. 3, Supplementary Table S1 and Supplementary Fig. S1. The fractions of the ejaculate used to prepare the seminal doses did not influence total and progressive motility, VSL, VAP, ALH, LIN, STR and WOB parameters (Fig. 3, Supplementary Table S1 and Supplementary Fig. S1). However, a significant effect was observed for VCL and BCF (Supplementary Table S1). VCL was higher in F1 than in F3 (*P* = 0.03), whereas F2 group did not show a significant difference compared to both groups. The BCF was greater in F1 than in F2 and F3 group (*P* = 0.04), without a significant difference between F2 and F3. Moreover, viability, acrosome integrity, mitochondrial activity, and DNA fragmentation of the sperm, did not show significant differences between the experimental groups (Supplementary Table S1). Any of the parameters analyzed showed interaction between time and treatment (Fig. 3 and Supplementary Fig. S1).

On day 5, the thermo-resistance test (TRT) was performed with spermatozoa from different accumulative ejaculate fractions (Table 1). Any of the sperm parameters evaluated showed differences between the groups (*P* > 0.05), except the progressive motility that was higher in F2 and F3 than in F1 (*P* = 0.04) (Table 1).

**Table 1:** The effect of thermo-resistance test (TRT) on boar sperm from different ejaculated fractions (F1, F2, F3) after 5 days of seminal doses refrigeration (∼16 °C).

	Experimental groups			*P*-value
	F1-TRT	F2-TRT	F3-TRT	
Total motility (%)	37.26 ± 4.81	48.74 ± 4.86	53.21 ± 4.73	0.07
Progressive motility (%)	19.37 ± 3.37^a^	28.00 ± 3.12^b^	27.37 ± 2.89^b^	0.04
VCL (µm/s)	37.32 ± 3.61	39.63 ± 3.64	40.16 ± 2.88	0.69
VSL (µm/s)	18.00 ± 3.06	20.00 ± 3.18	19.37 ± 2.42	0.64
VAP (µm/s)	24.32 ± 3.18	25.79 ± 3.23	25.95 ± 2.51	0.75
ALH (µm)	1.26 ± 0.10	1.32 ± 0.11	1.26 ± 0.10	0.92
LIN (%)	44.16 ± 3.56	46.63 ± 3.41	46.63 ± 2.92	0.57
STR (%)	66.42 ± 3.39	71.79 ± 2.72	71.11 ± 2.08	0.16
WOB (%)	61.00 ± 3.21	63.16 ± 2.73	63.47 ± 2.31	0.64
BCF (Hz)	4.26 ± 0.46	4.74 ± 0.48	5.11 ± 0.33	0.15

Values within a row with different superscripts (^a,b^) differ significantly between procedures (F1-TRT, F2-TRT, F3-TRT) at *P* < 0.05. Data are provided as mean ± SEM.

When we mimicked the in vivo conditions, sperm were incubated in vitro, after 5 days of storage, with UF at 38.5 ºC for 3 h. Sperm quality from each experimental group (F1, F2 and F3) was compared with its corresponding after incubation with UF, and they showed significant differences in several parameters (Tables 2, 3 and 4; Supplementary Fig. S2). In general terms, sperm from F1 fraction was the most influenced having 6 negatively affected parameters and two tendencies (for a total of 14 parameters evaluated), while F2 had 4 reduced parameters and one tendency, and F3 had 3 reduced parameters and one tendency (Supplementary Fig. S2). Independently of the fraction used, total motility, ALH, LIN, STR, WOB and viability of the sperm were always affected (positively or negatively) after UF incubation (Supplementary Fig. S2). Total sperm motility significantly decreased for each ejaculate fraction after being incubated with UF (*P* < 0.001). This reduction was more pronounced in the F1 group than in F2 and F3 (37.24%, 29.14% and 27.41%, respectively; the percentage was calculated as follows: (UF-motility × 100/F-Motility) -100). Regarding ALH, it also decreased after seminal doses incubation with UF (*P* < 0.01). LIN, STR and WOB were higher in the groups incubated with UF than those before the incubation (*P* < 0.001). Moreover, VCL and BCF were higher in F1 than F1 + UF (*P* = 0.002 and *P* = 0.007, respectively), but no significant differences were observed for F2 and F3.

**Table 2:** The effect of uterine fluid (UF) incubation on boar spermatozoa from F1 ejaculate fraction after 5 days of storage (∼16 °C). Both groups (F1 and F1 + UF) were incubated for 3 h (38.5 ºC) before the analysis.

	Experimental groups		*P*-value
	F1	F1 + UF	
Total motility (%)	90.32 ± 1.72^a^	55.74 ± 3.23^b^	< 0.0001
Progressive motility (%)	38.89 ± 2.95	33.16 ± 2.40	0.31
VCL (µm/s)	65.42 ± 5.03^a^	53.63 ± 3.04^b^	0.002
VSL (µm/s)	24.68 ± 1.22	29.84 ± 1.90	0.23
VAP (µm/s)	41.58 ± 2.28	39.21 ± 1.92	0.06
ALH (µm)	2.16 ± 0.12^a^	1.47 ± 0.12^b^	0.0004
LIN (%)	40.11 ± 2.68^a^	56.21 ± 2.17^b^	< 0.0001
STR (%)	60.26 ± 2.84^a^	76.05 ± 2.03^b^	0.0001
WOB (%)	65.21 ± 1.79^a^	73.26 ± 1.65^b^	0.002
BCF (Hz)	7.53 ± 0.16^a^	7.11 ± 0.17^b^	0.007
Viability (%)	89.58 ± 1.02^a^	84.63 ± 1.07^b^	0.03
Acrosome integrity (%)	93.26 ± 0.45^a^	89.68 ± 0.84^b^	0.006
Mitochondrial activity (%)	90.47 ± 0.71	84.05 ± 2.53	0.06
DNA fragmentation (%)	0.84 ± 0.38	0.53 ± 0.16	0.38

Values within a row with different superscripts (^a,b^) differ significantly between procedures (F1, F1 + UF) at *P* < 0.05. Data are provided as mean ± SEM.

**Table 3:** Effect of uterine fluid (UF) incubation on boar spermatozoa from F2 ejaculate fraction after 5 days of storage (∼16 °C). Both groups (F2 and F2 + UF) were incubated for 3 h (38.5 ºC) before the analysis.

	Experimental groups		*P* value
	F2	F2 + UF	
Total motility (%)	89.89 ± 1.38^a^	61.95 ± 3.62^b^	< 0.0001
Progressive motility (%)	35.74 ± 3.37	36.84 ± 2.50	0.94
VCL (µm/s)	58.95 ± 1.68	57.89 ± 4.91	0.59
VSL (µm/s)	22.21 ± 1.43^a^	30.74 ± 2.32^b^	0.02
VAP (µm/s)	38.47 ± 0.88	42.26 ± 2.58	0.74
ALH (µm)	2.11 ± 0.11^a^	1.58 ± 0.12^b^	0.0007
LIN (%)	37.32 ± 2.47^a^	54.74 ± 3.00^b^	< 0.0001
STR (%)	56.11 ± 3.02^a^	72.74 ± 2.74^b^	< 0.0001
WOB (%)	65.95 ± 1.58^a^	74.26 ± 1.78^b^	0.0001
BCF (Hz)	6.95 ± 0.21	6.89 ± 0.21	0.49
Viability (%)	90.32 ± 0.66^a^	85.63 ± 1.31^b^	0.01
Acrosome integrity (%)	93.00 ± 0.51^a^	90.21 ± 0.78^b^	0.02
Mitochondrial activity (%)	89.47 ± 0.77	85.53 ± 1.87	0.06
DNA fragmentation (%)	0.53 ± 0.12	0.53 ± 0.18	0.75

Values within a row with different superscripts (^a,b^) differ significantly between procedures (F2, F2 + UF) at *P* < 0.05. Data are provided as mean ± SEM.

**Table 4:** Effect of uterine fluid (UF) incubation on boar spermatozoa from F3 ejaculate fraction after 5 days of storage (∼16 °C). Both groups (F3 and F3 + UF) were incubated during 3 h (38.5 ºC) before the analysis.

	Experimental groups		*P* value
	F3	F3 + UF	
Total motility (%)	88.68 ± 1.14^a^	63.47 ± 3.21^b^	0.0002
Progressive motility (%)	38.68 ± 2.66	38.68 ± 2.49	0.44
VCL (µm/s)	59.16 ± 3.48	53.84 ± 2.91	0.15
VSL (µm/s)	23.42 ± 1.15^a^	29.63 ± 1.84^b^	0.01
VAP (µm/s)	38.63 ± 1.55	38.26 ± 1.68	0.47
ALH (µm)	2.05 ± 0.12^a^	1.58 ± 0.12^b^	0.02
LIN (%)	40.58 ± 1.86^a^	55.00 ± 2.38^b^	0.0005
STR (%)	61.05 ± 2.22^a^	76.00 ± 2.08^b^	0.002
WOB (%)	66.32 ± 1.45^a^	71.74 ± 1.65^b^	0.0006
BCF (Hz)	7.00 ± 0.19	7.16 ± 0.30	0.52
Viability (%)	90.16 ± 0.52^a^	86.84 ± 0.79^b^	0.004
Acrosome integrity (%)	93.11 ± 0.45	90.26 ± 0.77	0.06
Mitochondrial activity (%)	87.84 ± 1.50	86.42 ± 1.05	0.09
DNA fragmentation (%)	1.05 ± 0.49	0.47 ± 0.14	0.09

Values within a row with different superscripts (^a,b^) differ significantly between procedures (F3, F3 + UF) at *P* < 0.05. Data are provided as mean ± SEM.

The viability of sperm was also reduced after being incubated with UF whichever was the ejaculated fraction used (*P* < 0.01). Furthermore, acrosome integrity decreased in F1 + UF and F2 + UF with respect to F1 and F2 (*P* < 0.01), and a tendency was observed for the F3 group (*P* = 0.06). Likewise, sperm mitochondrial activity showed a statistical tendency in F1 and F2 groups (*P* = 0.06) but not for F3 (*P* > 0.05). DNA fragmentation did not show a significant difference after UF incubation in any of the analyzed groups (*P* > 0.05).

## Discussion

SP is an important part of the boar ejaculate in both, volume and composition. However, during seminal doses preparation for further use in AI, the SP is highly diluted because of the extender added^4^ to reach an adequate sperm concentration and dose volume. The addition of the last fractions of the ejaculate may solve the high rate of SP dilution, however, it has been controversial in the swine industry. Our results have demonstrated that sperm metabolic rates (day 1) and sperm quality during preparation and storage of the seminal doses (up to 5 days) have similar performance independently of the ejaculate fraction included. Although, the presence of SP from F2 and F3 fractions in the seminal doses after short-term storage helps the sperm to keep their function when subjected to a heat stress (TRT) or after being incubated with UF (mimicking the in vivo conditions).

One of the keys to keep sperm motility high is the presence of energetic compounds in the media surrounding the cells^24^ based on ATP production. Once ejaculated, sperm get in contact with the SP rich in energetic substrates such as fructose, glucose, and sorbitol^12^, which promotes the vigorous movement of the sperm. Our results indicate that sperm metabolism after ejaculation (pure semen), analyzed in terms of OCR and ECAR, was similar between experimental groups, supporting that SP contains enough energetic compounds to maintain similar metabolic sperm rates regardless of the ejaculate fraction during at least the first hours after ejaculation (~ 12 h). After semen dilution to prepare the seminal doses, the proportion of SP in each group was different, accordingly with a previous study^4^, so the volume of the extender added differs, being greater in F1 than in F2 and F3 (the group with the highest rate of SP and lowest volume of extender added). In any case, the metabolic rates analyzed were equal between groups and between pure and diluted semen, which indicates that the extender supplies the energetic substrates after SP dilution in the same way in all of the groups. Additionally, F2 and F3 did not show any difference from F1, thanks to the presence of a larger volume of SP, and consequently, a greater buffering capacity, equalizing the metabolic activity among the different accumulative fractions of the ejaculate.

Moreover, it was interesting to analyze the OCR during basal and maximal respiration to have a more complete view of sperm metabolism. The OCR during both types of respiration was similar between the groups, without difference between pure and diluted semen. Furthermore, basal respiration was close to the maximal respiration induced by the FCCP injection, resulting in a low spare respiratory capacity. This could suggest that sperm mitochondria from different accumulative ejaculate fractions work at their maximal capacity for ATP production. This fact could be supported by the presence of the same metabolites involved in energy production and lipid synthesis within the different ejaculate fractions, changing only in concentration^25^.

According to the sperm metabolism results, our study did not show any differences in sperm quality parameters (motility, viability, acrosome status, mitochondrial activity; DNA fragmentation) between the experimental groups during semen storage. Although, previous studies showed a controversial effect of SP, highlighting the worst quality and resistance to liquid-storage of sperm from the post-spermatic fraction^26^, whereby in AI-centers the sperm-rich fraction is commonly used for seminal doses preparation. Despite this, the inclusion of SP from all the ejaculate fractions has recently been seen to lead to the maintenance of sperm functionality and the same reproductive outcomes, as when using only the rich fraction^4,27^, supporting our results. Nevertheless, SP is known to have a controversial effect on sperm quality during storage, which can be influenced by several factors such as the dilution rate and final sperm concentration. In fact, during studies in which a low sperm concentration was used (< 18 × 10^6^ sperm/ml), a decrease in sperm quality was observed^28^. In our case, a concentration of ~ 30 × 10^6^ sperm/ml was used, so a different dilution rate was applied, depending on the fraction, being characterized by a different SP volume. In fact, the whole ejaculate (F3) has a greater amount of SP compared to the sperm-rich fraction (15% vs. 8%)^4^, which can be useful to maintain sperm functionality. Thus, we could deduce that SP from the bulk ejaculate is not harmful to boar sperm storage. In particular, during ejaculation, several proteins from different SP fractions adhere to the sperm surface^16^ and remain attached as well during storage^28^. Besides, SP from the post-spermatic fraction stands out from the other fractions for an increased expression of some proteins such as alpha-enolase or alkaline phosphatase, involved in maintaining sperm motility^9,29^. Therefore, these proteins would continue to carry out their function of stabilizing the sperm membrane.

The final goal of seminal doses is to be used during the AI, in which spermatozoa are deposited in the female reproductive tract. Here, sperm will reside for hours at a temperature of 38.5 °C and be surrounded by UF before reaching the oviduct. Thus, it is interesting to understand how sperm from different accumulative fractions would react to this situation after several days of storage at 16 ºC. For this, sperm from each type of dose was subjected to a TRT. Sperm cells behaved similarly except for the progressive motility which was greater when several fractions (F2 or F3) were included in the dose compared to the rich fraction only (F1). Sperm motility is known to be supported by the ATP concentration, which can be altered by different factors such as heat stress. In fact, prolonged exposure to high temperatures triggers the dephosphorylation of Ser21, activating the protein glycogen synthase kinase 3, which inhibits ATP production^30^. Also, other proteins may be involved in decreasing the progressive motility, such as spermadhesins (AWN, PSP-I/PSP-II), originated from the vesicular glands^31^, and present in the bulk ejaculate^9^. In particular, sperm cells from the post-spermatic fraction absorb a high concentration of proteins from SP like the heterodimer PSP-I/PSP-II^9,32^ and the heat shock proteins (especially HSP90), both involved in increasing thermal resistance, playing a key role in the maintenance of sperm motility and viability^33^. Therefore, it could be hypothesized, that these proteins not only offer protection at low temperatures but also could play the same role in heat stress protection.

Within the uterus sperm contact with UF, exerting a negative effect on spermatozoa that can be mitigated by the presence of SP^18^. In the present study, the sperm quality decreased in the presence of UF independently from the SP fraction. However, sperm from F3 and F2 were those in which the quality parameters were less affected. This fact could be explained by the higher volume and different composition of SP in F3 and F2 than in F1^4^. Actually, SP concentration in F3 (~ 15%) is close to the concentration used in a previous study in which 20% of SP (only from the rich fraction) was able to mitigate the negative effect of UF^18^. The effect of UF in some kinetic parameters (LIN, STR, and WOB) was positive in all three experimental groups, moreover, a positive effect on VSL in F2 and F3 groups (with no changes in F1) is worth mentioning. VSL has been positively related to ATP content per sperm in mammals^34^, and ATP is considered to be responsible for the generation of sperm forward motility. In our case, sperm motility decreased in all experimental groups after UF, but this drop was less noticeable in F2 and F3 when compared with the motility without the UF presence. So, the sperm of the F2 and F3 groups could contain more ATP and in consequence a higher VSL and less loss of sperm motility. As mentioned above, sperm motility strictly depends on ATP concentration. How much ATP sperm cells are able to produce may depend on the content of extracellular vesicles present in the SP. These vesicles come from different accessory glands for which they are present in all fractions and infiltrate the sperm membrane by transferring their content (i.e., proteins, small non-coding RNA) and maintaining sperm function^35^. Between these vesicles, CD44 has been described to play a role in the sperm interaction with female reproductive fluids, however, it is present in a similar cargo between the fractions^35^. Considering this, we can hypothesize that sperm from F2 and F3, containing a higher amount of SP than F1, also have a greater quantity of extracellular vesicles and it can result in higher ATP production. Moreover, since the different fractions of the ejaculate differ in protein and lipid composition, and the higher concentration of proteins is in SP from the post-spermatic fraction, this SP could protect sperm from the uterine environment^36^. Thus, the inclusion of SP from all ejaculate fractions may be beneficial to sperm functionality under certain conditions. In general terms, changes observed in sperm kinetic parameters after UF incubation may be attributable to the viscosity of the surrounding fluid. However, the addition of UF in the proportion as in the present work does not influence the viscosity of the media^18^.

To sum up, it seems clear that our results indicate, that sperm keep their function after short-term storage, independently of the ejaculate fraction/s included in the seminal doses; however, when the semen was subjected to thermal stress or incubation with UF, a detrimental effect on sperm was shown in all groups, but being more noticiable in F1 fraction. This disparity between groups was not observed in the reproductive performance of a previous study^4^. Two possibilities, not mutually exclusive, could explain these results. First, the reproductive performance obtained in the mentioned study was carried out after 3 days of storage, while the present study was performed after 5 days. This difference during the storage period could increase the sperm sensibility to external processes such as TRT or UF incubation, being more pronounced in the group lacking some components of the SP such as in the F1 group. Second, during commercial porcine AI a high number of sperm are deposited in the female genital tract (~ 2–4 × 10^9^ sperm/60–90 ml), therefore any negative effect influencing semen in their trajectory may be masked, because of the high number of spermatozoa reaching the site of fertilization. This being said, further studies using a reduced number of sperm should be performed in order to elucidate differences in vivo after AI as has been observed in in vitro conditions.

## Conclusions

Overall, our results show that sperm metabolism is not influenced by the ejaculate fraction, being the compounds of pure and diluted semen enough to equally maintain the bioenergetics of the sperm between accumulative fractions at least during the first hours after ejaculation. In addition, we have found that sperm quality during semen storage for up to 5 days did not change, even though the origin of SP has proceeded from different ejaculate fractions. Nevertheless, the behavior of the sperm after storage was modified depending on the ejaculated fraction. When subjected to thermal stress and UF incubation, seminal doses prepared with the whole ejaculate being the least affected. The results can also support the hypothesis, that the different components of the SP in the milieu mimicking the uterus (thermal stress or UF) exert an influence over the spermatozoa. All these findings have important implications. From a basic science point of view, it shows metabolic and storage behavior of sperm, and their response to thermal and fluid (UF) stressors from different accumulative ejaculate fractions. From a practical point of view it is important as well for the swine industry, showing the impact of seminal doses preparation and storage on a routine workflow in AI-centers. Although there is evidence showing sperm features in response to SP, further research is necessary to fully understand the interaction between SP compounds and the sperm, and the impact of the milieu once sperm is deposited in the female genital tract.

## Materials and methods

### Ethics statements

All the procedures for this study were approved by the Ethical Committee of the University of Murcia on 1 June 2020 (reference project PID2019-106380RB-I00 and ethical committee reference 567/2019). All methods were performed in accordance with the relevant guidelines and regulations. The study is reported in accordance with ARRIVE guidelines.

### Experimental design

**Figure 4:**
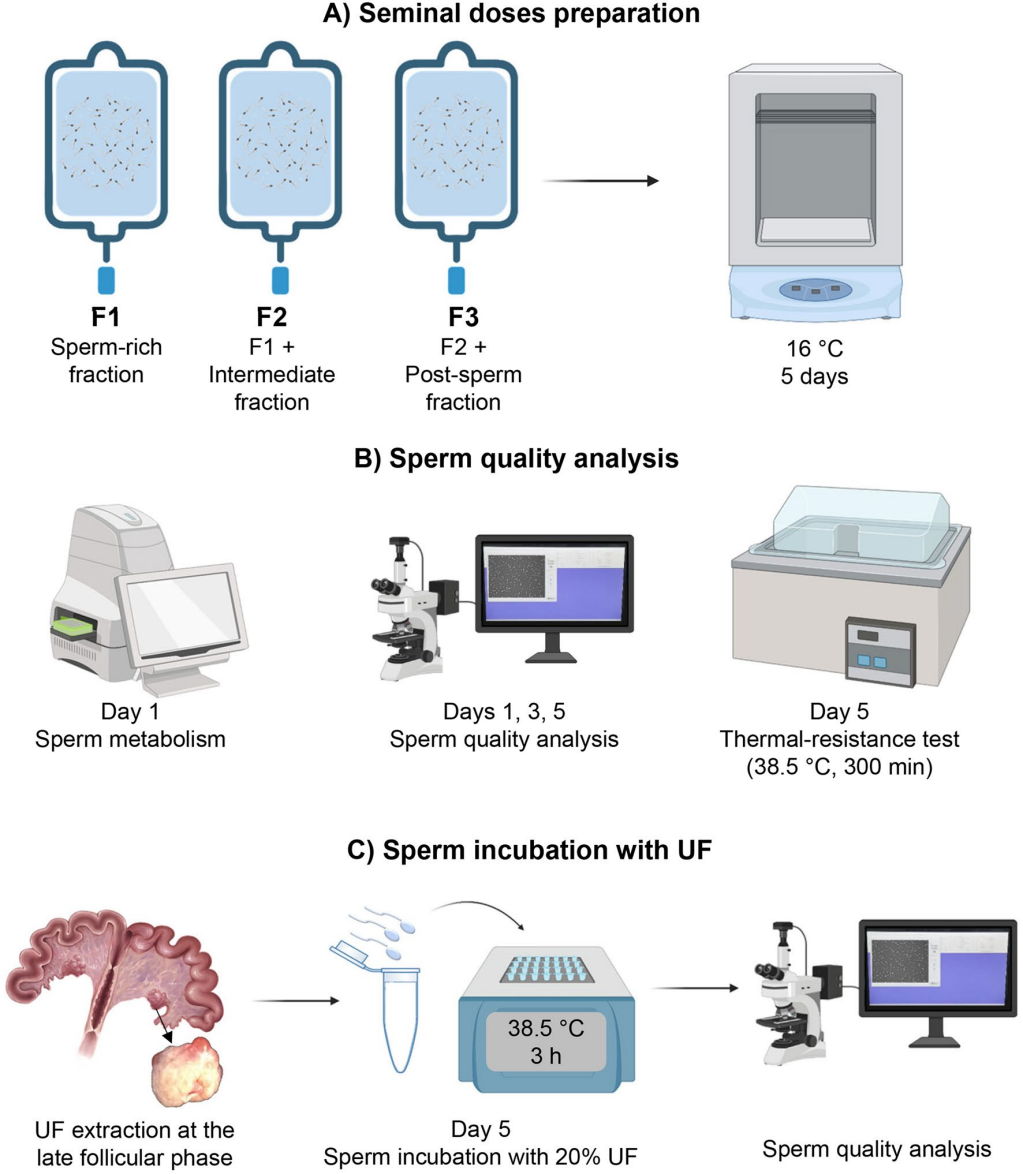
Graphic representation of the experimental design. (**A**) Three types of seminal doses were prepared (F1: sperm-rich fraction; F2: F1 + intermediate fraction; F3: F2 + post-sperm fraction) and stored at 16 °C for up to 5 days. (**B**) Evaluation of sperm functionality (I): on day 1 of conservation, the sperm metabolism (pure and diluted semen) was evaluated by the Seahorse analyzer; on days 1, 3 and 5 of seminal doses refrigeration, the sperm quality (motility and kinetics, viability, acrosome integrity, mitochondrial activity, DNA fragmentation) was evaluated; on day 5 the TRT was performed by incubating seminal doses at 38.5 °C during 300 min. (**C**) Evaluation of sperm functionality (II): uterine fluid (UF) from the late follicular phase was extracted. On day 5 semen from each seminal dose was incubated with 20% of UF for 3 h, and sperm quality analysis was performed. The figure was partially created on https://biorender.com (accessed on 8 June 2022).

Three experimental groups containing different ejaculated fractions were analyzed (Fig. 4): F1 (spermatic-rich fraction); F2 (spermatic-rich fraction plus intermediate spermatic fraction); F3 (spermatic-rich fraction plus intermediate spermatic fraction plus post-spermatic fraction). On day 1 (∼12 h after ejaculate extraction), sperm metabolism (OCR and ECAR) from pure semen and seminal doses was analyzed (n = 8 for each condition and experimental group). Then, sperm motility (total and progressive), kinetic parameters, viability, acrosome status, mitochondrial activity, and DNA fragmentation were evaluated on days 1, 3 and 5 of seminal doses refrigeration (n = 19 for each experimental group) (Fig. 4). Before evaluation, the seminal dose was homogenized and an aliquot for each semen sample was warmed in a heat block (Accu Block®, Labnet International, Inc., New York, USA) at 38.5 °C for 10 min. On day 5, an aliquot of 10 ml from each sample (n = 19) was subjected to the thermal-resistance test (TRT; 300 min at 38.5 °C), and thereafter sperm motility (total and progressive), kinetic parameters, viability, acrosome status, mitochondrial activity, and DNA fragmentation were evaluated (Fig. 4). Finally, on day 5, sperm from seminal doses were incubated with UF. Specifically, 20% of UF was added to an aliquot from each experimental group (F1, F2 and F3) in a final volume of 1 ml. All the samples (with and without UF) were incubated at 38.5 °C in a heat block, and after 3 h sperm motility (total and progressive), kinetic parameters, viability, acrosome status, mitochondrial activity, and DNA fragmentation were evaluated (Fig. 4). A total of 11 replicates were performed for this experiment.

### Animals

Six boars (Pietrain German Genetics; 30.83 ± 2.63 months of age) housed in a commercial AI-center (Sergal Gestió Ramadera, Catalonia, Spain) were included in this study. Animals were housed in individual pens (sawdust or straw bed; according to the European Commission Directive for Pig Welfare) within the same building and were fed by commercial feed (pellets). Temperature levels were maintained automatically by a climate control system, ensuring that the room’s temperature remained within the range of 18–22 °C. From 7 months of age, the basal feed ration (2 kg) was increased based on the body condition of the boars (maximal feed ration of 3.5 kg/day). Water was available ad libitum. Boars were dewormed twice per year and there were parvovirus and erysipelas vaccinations. The frequency of semen collection was ∼1.5 times per week with an interval of 4–6 days between collections.

### Uterine fluid (UF) collection and preparation

The UF, obtained from female genital tracts at the slaughterhouse (El Pozo S.A., Alhama de Murcia, Murcia, Spain), was provided by EmbryoCloud (NaturARTs®, Murcia, Spain). The oestrus cycle stage corresponded with the late follicular phase [periovulatory follicles (8-11 mm Ø)] based on the appearance of the ovary. The female genital tracts were transported to EmbryoCloud laboratory at room temperature within 30 min after collection and the UF was extracted, centrifuged twice at 7200 g for 10 min at 4 °C to remove debris, and finally stored at -80 °C until use. For the experiments, a pool of UF from 3 different females (represented in the same proportion) was used.

### Ejaculate collection and dilution

A total of 57 ejaculates from the six proven fertility boars (~ 9 ejaculates per boar) were collected by the gloved-hand method. The pre-spermatic fraction was discarded, and the following ejaculated fractions were collected: 1) spermatic-rich fraction (F1) (n = 19), 2) spermatic-rich fraction plus intermediate fraction (F2) (n = 19), and 3) spermatic-rich fraction plus intermediate fraction plus post-spermatic fraction (F3) (n = 19). Then, the concentrations of the samples were calculated for each fraction by Spermacue photometer (Minitϋb, Germany). After that, ejaculated sperm fractions were diluted in a commercial extender (Androstar® Plus, Minitube International, Tiefenbach, Germany) at 32 °C to reach a final concentration of ~ 33 × 10^6^ sperm cells per ml. Then, each sample was packaged in plastic bags with 2000 × 10^6^ sperm/60 ml and incubated at room temperature (18–22 °C) for 120 min. Finally, seminal doses were refrigerated at 16 °C till the time of evaluation.

### Evaluation of spermatozoa motility and kinetic parameters

The Computer Assisted Semen Analysis (CASA) was used for the evaluation of spermatozoa motility by ISAS® software (PROiSER R + D S.L., Valencia, Spain) connected to a phase-contrast microscope (negative-pH 10 × objective; Leica DMR, Wetzlar, Germany) and a digital camera (Basler Vision, Ahrensburg, Germany). The samples were warmed at 38.5 °C during 10 min. Then, a 4 µl drop of the sample was placed in a prewarmed (38.5 ºC) chamber (20 microns Spermtrack® chamber, Proiser R + D, SL; Paterna, Spain) and at least three fields per sample were recorded. CASA setting parameters used were 25 frames per second and particle size area between 10 and 80 µm^2^. Spermatozoa trajectory was classified into three categories: slow (10–25 µm/s), intermediate (> 25–45 µm/s), and rapid (> 45 µm/s). Total motility (%), progressive motility (%), and the kinetic parameters as curvilinear velocity (VCL, µm/s), average path velocity (VAP, µm/s), straight-line velocity (VSL, µm/s), amplitude of lateral head displacement (ALH, µm), linearity of the curvilinear path (LIN, ratio of VSL/VCL, %), straightness of the average path (STR, ratio of VSL/VAP, %), wobble coefficient (WOB, %), and beat-cross frequency (BCF, Hz) were analyzed.

### Evaluation of spermatozoa viability

The percentage of viable spermatozoa was determined by evaluating membrane integrity with propidium iodide (PI; P-4170 Sigma-Aldrich®, Madrid, Spain). The staining solution was prepared with 50 µl of PI (500 µg/ml) in 10 ml PBS (Phosphate Buffer Solution) without Ca^2+^ and Mg^2+^ (Sigma-Aldrich®, Madrid, Spain). Sperm samples were incubated with a PI solution for 10 min at room temperature under darkness. Samples were evaluated under fluorescence microscope (40 × objective; Leica® DM4000 Led, Wetzlar, Germany, 495/520 nm) and at least 200 cells were counted per sample. For the evaluation, spermatozoa without fluorescence were classified as live, and sperm with red-stained heads were classified as dead.

### Evaluation of spermatozoa acrosome status

Sperm acrosome status was evaluated by *Arachis hypogaea* lectin (PNA-FITC, Sigma Aldrich®, Madrid, Spain). The staining solution was prepared with 100 µl of PNA-FITC (200 µg/ml) in 10 ml of PBS without Ca^2+^ and Mg^2+^. Sperm samples were incubated with PNA-FITC solution for 10 min at room temperature under darkness. Samples were evaluated under fluorescence microscope (40 × objective; Leica® DM4000 Led, Wetzlar, Germany, 495/520 nm) and at least 200 cells were counted per sample. Sperm were classified according to acrosome status into one of the following categories: 1) normal apical ridge: sperm acrosome without fluorescence; 2) damaged apical ridge: sperm acrosome showing green fluorescence.

### Evaluation of spermatozoa mitochondrial activity

Sperm mitochondrial activity was evaluated by JC-1 (5,5’,6,6’-tetrachloro-1,1’,3,3’-tetraethylbenzimidazolylcarbocyanine iodide; ThermoFisher Scientific Inc., MA, USA). The staining solution was prepared with 10 µl of JC-1 (0.017 µg/ml) in 10 ml of PBS without Ca^2+^ and Mg^2+^. Sperm samples were incubated with JC-1 solution for 30 min under darkness. Samples were evaluated under fluorescence microscopy (40 × objective; Leica® DM4000 Led, Wetzlar, Germany, 495/520 nm) and at least 200 cells were counted per sample. Sperm were classified into one of the following categories: (1) low mitochondrial membrane potential: sperm with green fluorescence in the midpiece; (2) high mitochondrial membrane potential: sperm with orange fluorescence in the midpiece.

### Evaluation of spermatozoa DNA fragmentation

Sperm DNA fragmentation was evaluated by a Halomax kit for Sus scrofa (Halotech DNA, Madrid, Spain) following the manufacturer’s instructions. Agarose was warmed at 100 °C for 5 min and then transferred to 37 °C for 5 min to equilibrate the temperature. Sperm samples were added to the vials with low melting agarose (1:2, v/v) and mixed at 37 °C. A 2 µl drop from each sample was placed onto a pre-coated slide provided in the kit, and left at 4 °C for 10 min to solidify, after that the coverslips were gently removed. The samples were treated first with lysis solution for 5 min and then distilled water for 5 min. Then, slides were dehydrated by sequential 70 and 100% ethanol baths, 2 min each, and air dried. Finally, slides were stained with red fluorescent stain (HT-RF S100, Fluored®, Halotech DNA, Madrid, Spain). Samples were evaluated under fluorescence microscope (40 × objective; Leica® DM4000 Led, Wetzlar, Germany, 495/520 nm) and at least 300 cells were counted per sample. Sperm were classified into one of the following categories: 1) unfragmented DNA: sperm with a small and compact halo of chromatin dispersion; 2) fragmented DNA: sperm with a large and spotty halo of chromatin dispersion.

### Thermal-resistance test (TRT)

The TRT is used to simulate the sperm exposition at 38.5 °C within the female genital tract until reaching the site of fertilization^37^. At day 5 of storage, an aliquot of 10 mL from seminal doses was incubated at 38.5 °C in a water bath for 300 min. Following the incubation, motility, viability, and acrosome integrity of the spermatozoa were assessed (as previously described) to test their resistance to temperature.

### Evaluation of spermatozoa metabolism

For the metabolic assay, a Seahorse XFe extracellular flux analyzer (Agilent Technologies, Inc., CA, USA) with 96-wells cell culture plate was used. The analysis was performed by using the Cell Mito Stress Assay kit (Agilent, Santa Clara, CA, USA). This kit contains three components that modulate cellular respiration, as indicated by the manufacturer’s instructions: (1) oligomycin: an ATP synthase inhibitor that decreases mitochondrial respiration; (2) carbonyl cyanide-4-(trifluoromethoxy)phenylhydrazone (FCCP): an uncoupling agent that collapses the proton gradient, which disrupts the mitochondrial membrane potential; (3) rotenone + antimycin A (Rot/AA): complex I and III of the electron transport chain inhibitors respectively, which shut down mitochondrial respiration. Then, the real-time Oxygen Consumption Rate (OCR, pmol/min), an indicator of mitochondrial respiration, and Extracellular Acidification Rate (ECAR, milli-pH/min), an indicator of glycolysis, of boar spermatozoa were measured through the analyzer. Moreover, the following bioenergetic parameters were analyzed: basal respiration representing the oxygen consumption before the oligomycin injection; maximal respiration representing the maximal oxygen consumption rate attained by adding the uncoupler FCCP, showing the maximum rate of respiration that cells can achieve; spare respiratory capacity which is the difference between maximal respiration and basal respiration, indicating the ability of the cells to respond to increased energy demand; and ATP production providing how fast cells work to produce ATP.

The protocol of the assay is depicted in Supplementary Fig. S3. One day before the assay, the sensor cartridge was hydrated with distilled water at 38.5 °C. On the day of the assay, distilled water was replaced with the calibration solution (Agilent Technologies, Inc., CA, USA), and the sensor cartridge was placed in the incubator at 38.5 °C without CO_2_ during 45–60 min. Then, the Seahorse XFe DMEM medium (pH = 7.4) (Agilent Technologies, Inc., CA, USA) was supplemented with sodium pyruvate (1 mM), glutamine (2 mM) and glucose (10 mM). Meantime, the components of the kit were prepared: 1) solution A: oligomycin 100 µM; 2) solution B: FCCP 50 µM; 3) solution C: Rot/AA 50 µM. Each solution was diluted in Seahorse XFe DMEM medium to reach the final concentration of 1.5 µM, 0.5 µM and 0.5 µM, respectively. Solution C was supplemented with Hoechst dye (25 mM; diluted 1:1000 to reach the final concentration of 2.5 µM per well) to perform the normalization of cell number after the assay^38^. Then, 20 µl of oligomycin, 22 µl of FCCP, and 22 µl of rot/AA were placed in the injection ports A, B and C of the utility plate, respectively. Once the sensor cartridge with the utility plate was ready, it was placed within the Seahorse XFe to start the calibration. Meantime, sperm concentration from each experimental group was calculated and adjusted at 1 × 10^6^ sperm/well in a 50 µl of volume and seeded in each well of an XFe96 Poly-D-Lysine microplate (Agilent Technologies, Inc., CA, USA). Four wells for each group were seeded per replicate (Fig. 1c-i) and four wells were left without cells to perform background corrections. Those were filled with DMEM medium, as well as the extra wells not filled with sperm cells. Then, the microplate was centrifuged at 300 g for 5 min with slow brake at room temperature (Thermo Scientific Heraeus® Multifuge® 3 Plus Centrifuge Series, Thermo Fisher Scientific Inc., MA, USA) to ensure the adhesion of cells to the bottom of the wells. Therefore, 130 µl of DMEM medium was gently added to each well, and the microplate was placed in the device. During the assay, the Seahorse XFe was kept at 38.5 °C. At the end of the assay, the microplate was placed in the incubator at 38.5 °C without CO_2_ for 30 min to allow the staining of sperm cells (Fig. 1c-ii). Then, the reading was performed using well-scan mode on the CLARIOstar Plus microplate reader (BMG Labtech, Ortenberg, Germany), as previously described^38^. The data were automatically normalized and analyzed by the Seahorse software. Finally, as a control assay, sperm viability was analyzed by collecting sperm cells from the wells and staining them with eosin/nigrosin. For each experimental group, 200 sperm were counted, the viability being similar between groups.

### Statistical analysis

When data was analyzed over time (days 1, 3 and 5), the statistical analysis was performed using the free statistical software, SAS University Edition (SAS, 2016). Fitting sphericity for repeated measurement was performed using the restricted likelihood ratio test between Huynh–Feldt (H-F) and unstructured (UN) covariance structures. If the difference between them (distributed under the null hypothesis as a χ2 with the difference between the degree of freedom, df) was greater than χ2 df, there was sphericity of data. All the motion parameters (total motility, progressive motility, VCL, VAP, VSL, LIN, STR, WOB, BCF), the percentage of alive spermatozoa, the percentage of spermatozoa with acrosome damage, the percentage of spermatozoa mitochondrial activity and DNA fragmentation showed sphericity of data and they were compared with the mixed model of SAS. The model included the experimental groups (F1, F2, F3), the time related to experimental groups (days 1, 3, and 5), and their interaction as the main effects, with replicates as a random effect. A first-order autoregressive covariance structure (AR1) was used to adjust the difference in data according to the differences over time. Regarding the metabolism, TRT, and incubation with UF evaluation, the statistical analysis was performed using the SPSS 24.0 software (IBM SPSS Inc., Chicago. IL, USA). The Shapiro-Wilks test was used to determine the normality of data. Then, one-way analysis of variance (ANOVA) was used to compare the experimental groups for the descriptive variables. Regarding metabolism analysis, each group before dilution (F1, F2, F3) was compared with its corresponding after dilution (F1 + UF, F2 + UF, F3 + UF) by using Student´s T-test. Data were expressed as the mean ± standard error of the mean (SEM). Differences were considered statistically significant when *P* ≤ 0.05 and a statistical tendency was considered when *P* ≥ 0.05 and ≤ 0.06.

### Supplementary Information

Supplementary Figure 1.

Supplementary Figure 2.

Supplementary Figure 3.

Supplementary Information.

Supplementary Legends.

## Data Availability

All data generated or analysed during this study are included in this published article and its supplementary information files.
