# Supplementary figures and images for: Impact of inclusion of post-spermatic ejaculate fraction in boar seminal doses on sperm metabolism, quality, and interaction with uterine fluid
Source: Sci Rep. 2023 Sep 14;13:15258. doi: 10.1038/s41598-023-42254-3 (PMC10502139; doi:10.1038/s41598-023-42254-3)

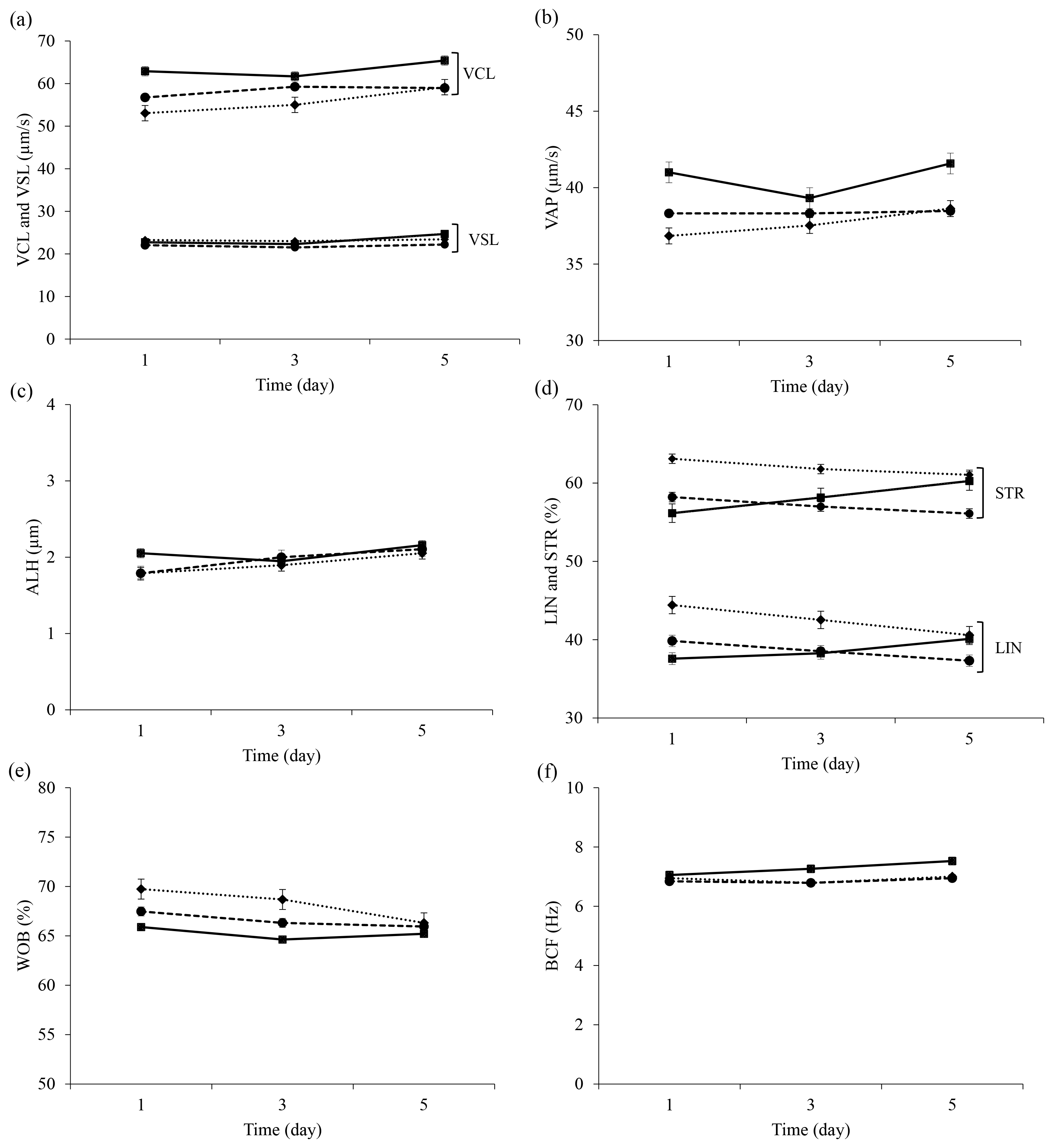

Supplement: Supplementary file 1 — Supplementary Figure 1. [file 41598_2023_42254_MOESM1_ESM.jpg]

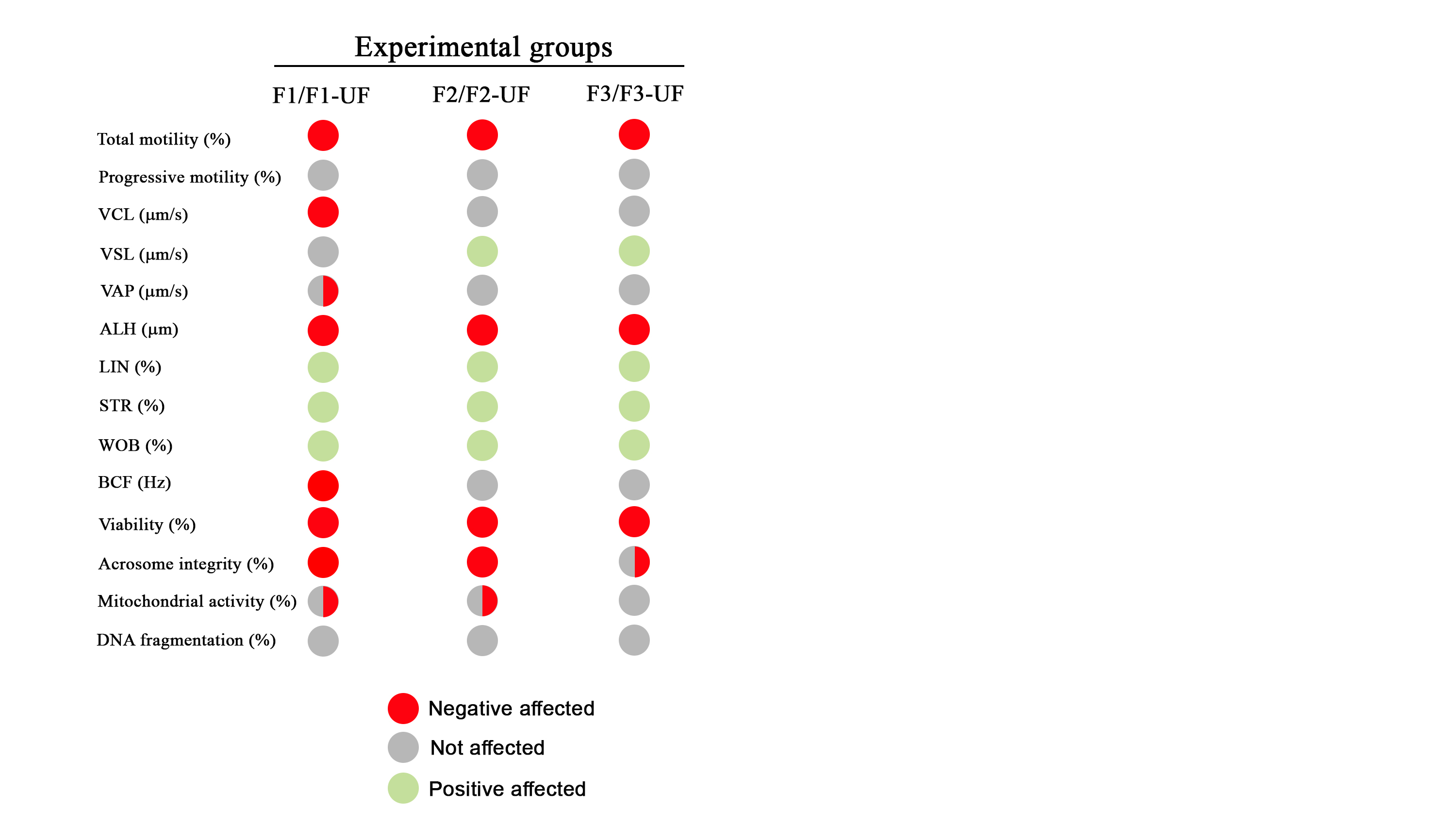

Supplement: Supplementary file 2 — Supplementary Figure 2. [file 41598_2023_42254_MOESM2_ESM.jpg]

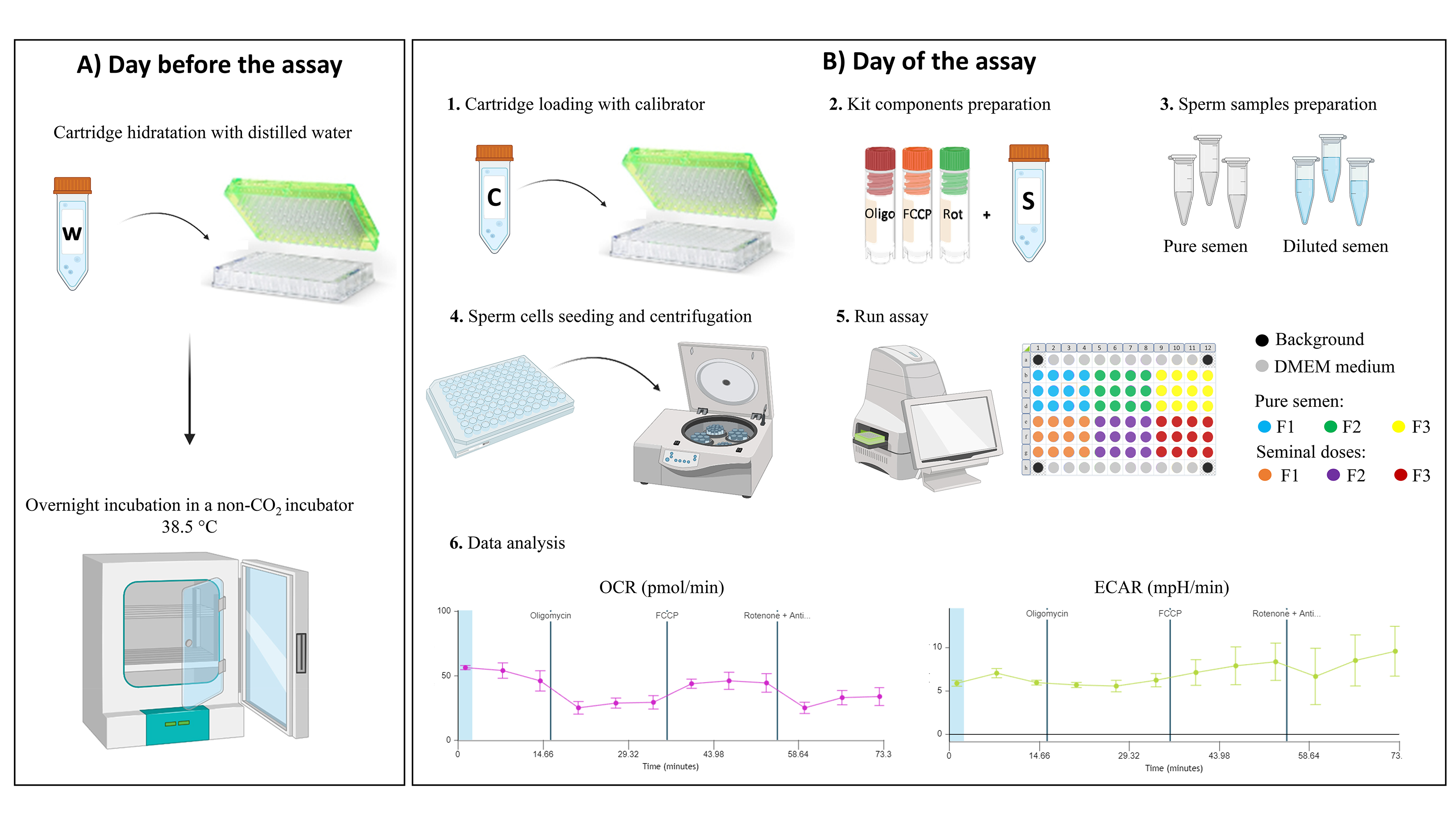

Supplement: Supplementary file 3 — Supplementary Figure 3. [file 41598_2023_42254_MOESM3_ESM.jpg]
