# Supplementary material for: Impact of inclusion of post-spermatic ejaculate fraction in boar seminal doses on sperm metabolism, quality, and interaction with uterine fluid
Source: Sci Rep. 2023 Sep 14;13:15258. doi: 10.1038/s41598-023-42254-3 (PMC10502139; doi:10.1038/s41598-023-42254-3)
Supplement: Supplementary file 4 — Supplementary Information. [file 41598_2023_42254_MOESM4_ESM.docx]

**Supplementary Table S1.** Quality parameters of boar spermatozoa from different accumulative ejaculated fractions stored for 5 days (analyzed at days 1, 3 and 5) at a refrigeration temperature of ∼16 °C: F1 (spermatic-rich fraction); F2 (F1 plus intermediate spermatic fraction); F3 (F2 plus post-spermatic fraction).

|  | **Experimental groups** | | |  |  |
| --- | --- | --- | --- | --- | --- |
|  | **F1** | **F2** | **F3** | ***P-*value** | **Pooled SEM** |
| Total motility (%) | 91.33 | 90.44 | 89.74 | 0.21 | 1.09 |
| Progressive motility (%) | 37.56 | 35.53 | 39.16 | 0.15 | 2.62 |
| VCL (µm/s) | 63.33^a^ | 58.32^ab^ | 55.74^b^ | 0.03 | 2.34 |
| VSL (µm/s) | 23.25 | 21.93 | 23.23 | 0.30 | 1.04 |
| VAP (µm/s) | 40.63 | 38.37 | 37.67 | 0.15 | 1.14 |
| ALH (µm) | 2.05 | 1.96 | 1.91 | 0.33 | 0.07 |
| LIN (%) | 38.65 | 38.56 | 42.51 | 0.13 | 2.10 |
| STR (%) | 58.19 | 57.10 | 61.98 | 0.11 | 2.33 |
| WOB (%) | 65.25 | 66.58 | 68.25 | 0.11 | 1.23 |
| BCF (Hz) | 7.28^a^ | 6.86^b^ | 6.91^b^ | 0.04 | 0.13 |
| Viability (%) | 92.09 | 92.33 | 91.84 | 0.46 | 0.56 |
| Acrosome integrity (%) | 94.77 | 94.40 | 94.37 | 0.24 | 0.37 |
| Mitochondrial activity (%) | 91.93 | 91.49 | 90.79 | 0.11 | 0.64 |
| DNA fragmentation (%) | 0.70 | 0.82 | 0.72 | 0.84 | 0.22 |

Values within a row with different superscripts (^a,b^) differ significantly between procedures (F3, F3+UF) at *P <* 0.05. Data are provided as mean ± pooled SEM.
