# Supplementary material for: Impact of inclusion of post-spermatic ejaculate fraction in boar seminal doses on sperm metabolism, quality, and interaction with uterine fluid
Source: Sci Rep. 2023 Sep 14;13:15258. doi: 10.1038/s41598-023-42254-3 (PMC10502139; doi:10.1038/s41598-023-42254-3)
Supplement: Supplementary file 5 — Supplementary Legends. [file 41598_2023_42254_MOESM5_ESM.docx]

**Impact of inclusion of post-spermatic ejaculate fraction in boar seminal doses on sperm metabolism, quality, and interaction with uterine fluid**

Luongo C^a^, Llamas-López PJ^b^, Garrappa G^a,c^, Rodríguez-Tobón E^a,d^, Grudzinska P^a^, García-Vázquez FA^a,e^

^a^Departamento de Fisiología, Facultad de Veterinaria, Campus de Excelencia Mare Nostrum, Universidad de Murcia, Murcia, España.

^b^Departamento de Tecnología Agroalimentaria, Universidad Miguel Hernández, Elche, España.

^c^Instituto de Investigación Animal del Chaco Semi-Arido (IIACS), Centro de Investigación Agropecuaria (CIAP), Instituto Nacional de Tecnología Agropecuaria (INTA), Tucuman, Argentina.

^d^Departamento de Biología de la Reproducción, Universidad Autónoma Metropolitana, Unidad Iztapalapa, Ciudad de México, México.

^e^Instituto Murciano de Investigación Biosanitaria (IMIB-Arrixaca), Murcia, España.

Corresponding author: Francisco Alberto García-Vázquez. Email: [fagarcia@um.es](mailto:fagarcia@um.es)

**Supplementary Figure S1.** Sperm kinetic parameters of boar sperm from different accumulative ejaculated fractions stored for 5 days (analyzed at days 1, 3 and 5) at a refrigeration temperature of ∼15 °C: F1 (spermatic-rich fraction) (–■–); F2 (F1 plus intermediate spermatic fraction) (--●‐‐); F3 (F2 plus post-spermatic fraction) (‧‧‧♦‧‧‧). (a) Curvilinear line velocity (VCL) and straight-line velocity (VSL), (b) average path velocity (VAP), (c) amplitude of lateral head displacement (ALH), (d) percentage linearity (LIN) and percentage straightness (STR), (e) percentage oscillation (WOB), (f) beat cross frequency (BCF). Data are provided as mean±SEM.

**Supplementary Figure S2**. Summary of the sperm parameters influenced by UF incubation. Red dots indicate that the evaluated parameter was negatively affected by UF incubation when comparing the counterpart ejaculate fraction (*P* < 0.05); green dots indicate that the evaluated parameter was positively affected by UF incubation (*P* < 0.05); grey dots indicate that the parameter was not affected (*P* > 0.05). Dots with grey/red color indicate a statistical tendency (when *P* ≥ 0.05 and ≤ 0.06) for negatively affected parameters after UF incubation.

**Supplementary Figure S3.** Graphic representation of boar sperm metabolism evaluation by the Seahorse analyzer Xfe96. F1= sperm-rich fraction; F2= F1 plus intermediate fraction; F3= F2 plus post-sperm fraction. OCR= oxygen consumption rate; ECAR= extracellular acidification rate. (A) The day before the assay the cartridge was hydrated with distilled water (W) and incubated overnight at 38.5 °C. (B) the day of the assay the cartridge was loaded with calibrator (C), and the kit components (oligomycin, FCCP, rotenone/antimycin) were prepared by diluting with Seahorse DMEM medium (S). Then, sperm samples (pure and diluted semen) were prepared, seeded into the plate and centrifugated. Finally, the assay was performed by Seahorse XFe96 analyzer, and OCR and ECAR were evaluated.
